# Beyond continental and African philosophies of personhood, healthcare and difference

**DOI:** 10.1111/nup.12393

**Published:** 2022-05-13

**Authors:** Elvis Imafidon

**Affiliations:** ^1^ Department of Religions and Philosophies, School of History, Religions and Philosophies SOAS University of London London UK

**Keywords:** African philosophy, continental philosophy, difference, embodied subject, Ubuntu

## Abstract

In this study, I explore the challenges that ideological hegemonies of personhood imbibed by nurses and other healthcare workers could pose for the nursing profession, particularly in terms of inhibiting the acknowledgment of difference. Dominant or hegemonic conceptions of personhood in particular spaces often consist of self‐contained ideas and essentialist ontologies and normativity of what it means to be a person, lack of which results in the denial of personhood and the othering as non‐person or sub‐person. The other as the residue of such self‐contained notions of personhood is most often denied the quality of care that the one who fits within such conceptions enjoy. For nurses and other healthcare workers to overcome such exclusionary tendencies in healthcare, they must overcome hegemonies and ideological dominance and be more open to alternative viewpoints and theories of personhood. I develop these lines of thought by focusing on the rich ideological traditions of Continental and African philosophies showing how exclusion takes place within these traditions based on conceptions of personhood and how such exclusion on the basis of difference impacts negatively on healthcare. I conclude by highlighting the need to go beyond hegemonic philosophies of personhood by decolonizing and demasculinizing healthcare, thereby allowing difference to flourish in an ecology of medical knowledge.

## 1 INTRODUCTION

Nursing in particular, and healthcare in general, are situations of encounters of selves and others. In nursing and healthcare situations, the caregiver and care‐receiver encounter their ontic and normative self or their ontic and normative other. Such encounter implies and presupposes one's assimilation of the ontology and normativity of the self or of personhood that permeates, or is entrenched in, one's horizon or place, which enables one to recognise and relate with a similar‐self, or recognise and differentiate oneself from a different‐other. The latter—the differentia self‐other encounter—sustains a self‐other binary often characterized not by a genuine hermeneutic ethic of the other, but by super‐altern—sub‐altern relationship, superiorist, supremacist, ethnocentric tendencies, and a differentia‐politics. This approach to the self and the other aggravates alterities and inhibits any genuine attempt to understand what really lies in between the self and the other: difference. With particular reference to healthcare, much of the exclusion, marginalization, and discrimination that happen in healthcare systems, I will show in what follows, emerge primarily from radical alterities built upon hegemonies of the self and of personhood and by implication, the non‐recognition or misrecognition of the difference that lies in‐between the self and the other and how such a difference can be a basis for building relationality, inclusivity, diversity and a rich and lively human experience and wellbeing.

Perhaps a good point to begin exploring and critiquing hegemonic philosophies of personhood is by conceptualizing what we mean by ‘philosophies of personhood’ and what makes them hegemonic. A philosophy of personhood is a grand and robust theory that interweaves ontic and normative features, qualities and characteristics that defines a human being—and in some cases a non‐human being (Chan & Harris, 2012)—as a person, what White (2013) calls the existential construct (the state of being inherent and essential to the human species) and the relational construct (the state of value defined by society) of the personhood of human species. There are two immediate implications of this understanding of a philosophy of personhood: first, not all human beings are human or persons, in the philosophical understanding of personhood, being a human being by possessing the anatomically or biologically defined corporeal and ontic features is not enough for being human, or to be a person as there are other latent ontic features as well as normative features one must possess to become a person. This first implication immediately draws our mind to some of the most fundamental and protracted issues in healthcare and bioethics such as the beginning of life debate, the debate on what sort of human being or life has value and is worth saving in a complicated healthcare situation, and debates about euthanasia and abortion. Although scientific cum medical explanations and discourses of these and related issues in medicine and bioethics may hesitate in acknowledging the philosophical presuppositions, particularly of personhood inherent in such explanations, debates, and discourses (Torchia, 2008), 'the contextual nature of bioethical dilemmas, the cultural embeddedness of moral systems, [and] the culturally pluralistic character of many bioethical problems,' (Muller, 1994; p. 448) show the strong connection between philosophical anthropology or ideas about human nature and personhood, bioethics and medicine. Second, being a person must be defined by a combination of ontic qualities including obvious and latent ones, and normative qualities often having to do with expected norms of behavior within specific contexts and places. These concerns about personhood are philosophical to the extent that they consist of ontological/metaphysical, epistemological, ethical, and logical issues surrounding the questions of personhood. Thus, the philosophy of personhood will be intensely concerned about such questions as what does it mean to be a person? How do we know that we are persons? How do we justify such knowledge claims? How ought a person to live and act as a member of a human community? Understood in this sense, therefore, philosophies of personhood indicate that there are several philosophical traditions, perspectives, and theories about these issues surrounding personhood. These philosophies emerge from different philosophical traditions and from different periods in human history.

In this study, I pay attention to two such traditions in exploring and analyzing the issues set forth above the Continental philosophical tradition and the African philosophical tradition. I begin by discussing an important foundational issue that is necessary to understand these philosophies of personhood by answering the question: why do philosophies of personhood matter at all for healthcare? I then proceed in the section that follows to look at the two philosophies of personhood, the Continental and the African and their impact on healthcare systems in the West and in Africa respectively. In Section 3, I examine the inherent hegemony and alterity in these philosophical traditions and their impact on healthcare. In the concluding section, I highlight approaches to take for more inclusive, decolonized and demasculinized healthcare systems to emerge.

## 2 WHY DO PHILOSOPHIES OF PERSONHOOD MATTER FOR HEALTHCARE?

Healthcare, in particular, human healthcare—recognizing immediately the limit of the concept of human healthcare as exclusionary to non‐human healthcare, is essentially structured around philosophical theories of personhood. The nature of the humanness or personhood of a patient—the question of who is a patient—has always been at the heart of medical and healthcare issues. Several issues that are at the heart of bioethical discourses on questions of moral responsibilities and moral issues in healthcare emerge from deeply embedded theories and understanding of human life, personhood, and wellbeing. Kasten (2008: 135) says that,

“Personhood” sounds like the purview of philosophers and theologians, the sort of topic that is more germane to those heady wine‐soaked evenings we enjoyed in college than to the white tiled corridors of the modern hospital. And yet the central questions posed by the concept – questions like “Who is a person and who is not?” “When does one begin – and cease – to be a person?” “Is there an enshrined view of personhood espoused by scientific medicine?” – are extremely relevant to clinical practice today. In fact, questions of personhood are so relevant that they comprise the most hotly debated political and ethical issues of our time. Stem cell research, abortion rights, end‐of‐life care, competency to stand trial, capacity to make decisions for one's self, the nature of mental illness – all presuppose a view of the human person.

James Marcum adds that

One of the most important components of any medical worldview is the nature of the patient. The patient is and should be the center and focus of a medical worldview, for without the patient there literally is no need for medicine. Consequently, a medical worldview is important for perceiving the patient, and that perception in turn shapes other components of a medical worldview, such as the nature of disease and health (2008: p. 61).

There is a growing and rich medical literature on the intrinsic connection between philosophical and theological theories of personhood and healthcare (Behrens, 2011, 2018; Chochinov et al., 2015; Imafidon, 2022; Ramsey, 1970; Thomasma et al., 2001; Tsai, 2008). This literature examines the pivotal and important roles concepts of persons and personhood play in medical care, the difficulty of theorizing personhood, and the impact of dominant perspectives of personhood in different healthcare contexts. For example, there have been some critical conversations around the limits of the consciousness model in healthcare (the presence of consciousness in a human body) and how concepts of personhood can enrich this dominant consciousness approach (Blain‐Moraes et al., 2018). There have also been discussions of how to broaden the understanding of evidence in evidence‐based medicine to incorporate more person‐based evidence (Henry et al., 2007; Raad, 2008) and how to make the understanding of, and approaches to, mental health more person‐centered (Matthews, 2001). Thus, while a number of scientists in general and healthcare practitioners, in particular, might not readily admit to the connections and co‐dependence of concerns in the sciences and medicine with those in the humanities in general and philosophy in particular, few would disagree that acknowledgment of personhood ought to be foundational within the culture of medicine (Chochinov et al., 2015). Therefore, ignoring philosophies of personhood, particularly those held mostly collectively, by the patients in specific contexts of healthcare would imply ignoring the very foundation of healthcare.

There are at least three specific reasons why philosophies of personhood are crucial for effective healthcare systems. The first concerns the understanding of healing. Consider, for example, the understanding of healing as a return. The need to heal a patient in this sense often implies that the patient falls short of an expected state of being (human), anatomically, mentally, or even spiritually (Matthews, 2001). The healing process is thus in a sense a process of returning a patient to such an ideal but essentially contested state of being, of personhood. It is a process of caring for and nurturing the patient in a manner that is hoped would rectify perceived past or future imbalances to the patient's personhood. When healing as return becomes difficult to attain in certain situations of medical care and health management, caring as a form of management of perceived imbalances, management that acknowledges that such an ideal state sought after in healing may not be reached, takes precedence over the quest to heal as a return. Thus, even in the understanding of caring and healing as helping patients to cope well, manage well or end well, there is the prioritizing of the dignity of the human person involved. In all caring and healing, therefore, there are obvious assumptions on ideal states of being human and being a person that shapes how such healing and caring happens. Holding tenaciously to certain assumptions about ideal states of personhood in healthcare as sacrosanct, hegemonic and essentialist would invariably imply excluding other perspectives from the healthcare system and by implication, the marginalization, and exclusion of those who hold such perspectives.

The second reason for the importance of philosophies of personhood for healthcare systems today is the renewed and increased interest in the last century in person‐centered care in healthcare systems. A person‐centric healthcare system, making persons the focal point of healing, caring for, and managing the health of such persons, invariably implies the need to take philosophies of personhood seriously. This is because person‐centered care is not merely patient‐centered care, where clinicians are morally obligated to put first the health needs and welfare of patients as medical objects. Understanding person‐centered care in such quite a narrow sense as exclusively patient‐centric, focussing solely on the person as an object of medical care with knowledge and experience that should be taken seriously in providing such care, raises quite a number of challenges as expected. For example, it fails to recognize that a person‐centric healthcare situation ideally should acknowledge and take seriously as moral equals all the persons involved, the clinician or health worker and the patient, not simply as medical subjects, but as persons with desires, values, cultures, unique circumstances and contexts, belief systems, lifeworld, and worldviews deserving of mutual respect, self‐care and reciprocal care (Buetow, 2016). Person‐centered healthcare is therefore a recognition of the plurality of value systems and normativity of personhood. A person‐centric healthcare system would not be sincerely person‐centered if it does not take philosophies of personhood seriously. 'Yet the anxiety in entering into these conversations regarding personhood is that it might take too long, detailing patient responses may be too onerous, or it could be emotionally evocative, for patients and [Healthcare Practitioners] However, failure to acknowledge personhood is often the root cause of patient and family dissatisfaction, and the reason why medicine is sometimes perceived as uncaring or emotionally abrasive.' (Chochinov et al., 2015: p. 975).

Finally, themes and issues in bioethics consist very importantly of themes and issues about the ethics of human life and personhood. There are three interrelated concerns of bioethics. First, in the most restrictive sense, bioethics as biomedical ethics consists of ethical issues that emerge from medical practice and medical research. Second, bioethics consists essentially of ethical issues emerging from the life sciences and technologies, including their impact on the environment. Third, in its most broad sense, bioethics consists of the ethics of the biosphere including moral issues and themes relating to human, non‐human and environmental aspects of the biosphere (Walker, 2006). Focussing specifically on bioethical concerns with personhood, a key focus in bioethical discourse is the beginning of, moral obligations to, and end of, human life. As Elvis Imafidon puts it,

The issue of when does human life begin takes a central and a crucial place in bioethics for two main reasons: (i) the [human] person occupies a central place in the scheme of things and fits rightly to what Martin Heidegger calls *Dasein*, one who occupies a here; and (ii), answers given to the question of the beginning of human life have direct moral implications on how the human being is treated particularly in the sphere of medicine with reference, for instance, to abortion, new reproductive technologies and euthanasia. Often debates on the beginning of human life are heated on whether the embryo or fetus can be said to be genuinely a living human being [a person].

The value of the person in healthcare, the moral permissibility or impermissibility of certain medical practices, research and technologies are often dependent on the understandings and conceptions of personhood that shapes a particular healthcare system. For example, Elvis Imafidon explains how differences in the ontologies and normativity of the person in Judeo‐Christian, Western‐secularist, and African philosophies result in the different perspectives on bioethical issues and varying commitments to the value of human life (Imafidon, 2014). Philosophies of personhood are thus crucial for bioethical discourses and for healthcare systems in general.

## 3 TWO PHILOSOPHIES OF PERSONHOOD

There are many rich and vibrant philosophical traditions around the globe, each with centuries of theorizing and philosophizing about reality, existence, knowledge, morality and other philosophical themes and issues. Although modern history saw the colonization of philosophy by the West and the formation of philosophy as a Western rather than human activity and practice (Park, 2013), the 20th/21st centuries has witnessed a revival of some sort of interests in the different philosophical traditions of the world. From ancient Greek philosophy, the golden age of Arab/Islamic philosophy, Egyptian philosophy, Confucian philosophy, Indian philosophy, and Persian philosophy, to sub‐Saharan African philosophy, there are fascinating and interesting theories and analyses of philosophical problems and themes including that of personhood. Interestingly, within broadly labeled philosophical traditions, such as Western philosophy, there are various approaches to philosophizing that permeate them such that such broad categorization is indeed not meant to convey sameness in thought. An example is the alleged differing approaches between Continental and British philosophies in the twentieth century and I will say a bit more about this shortly. Perhaps some volumes of books may not be enough to articulate, analyze and critique the concepts of a person entrenched into the different philosophical traditions of the world. For this reason, I focus here on two philosophical traditions only: Continental philosophy and African philosophy. Even so, it is an over‐ambitious agenda if my goal is to provide a robust exposition and analysis of these two philosophical traditions and their theories of personhood in the page of an article. What I may achieve at best in what follows is to highlight the most salient and widely held understandings in the existing literature of these traditions and their perspectives of personhood.

### 3.1 The continental philosophy of personhood and healthcare

Continental philosophy is not an easy philosophical tradition to define in a way that would be universally accepted—well, none of these traditions are easy to define this way. But there is much consensus that it is the philosophy of Europe, as distinct from the philosophy of the English‐speaking, Anglo‐American world, which was dominantly analytic philosophy (Glendinning, 2005). It covers a large period of philosophizing in Western philosophy, over 200 years, and consists of rich, diverse, and comprehensive philosophical ideas generally agreed to begin with Immanuel Kant's philosophy in the 1700s to the philosophical thoughts of French structuralists and postmodern philosophers such as Claude Levi‐Strauss, Jacques Lacan, Jacques Derrida and Michel Foucault in the late 1900s, but this history could be extended—or at least its roots deepened—as far back as the philosophy of the French philosopher, Rene Descartes, which were foundational to Continental philosophical thought. It, therefore, includes such philosophical movements and schools of thought as Existentialism, Hermeneutics, Phenomenology, Idealism and (Post)Structuralism (Critchley, 2001). The term itself was first coined and used by Oxbridge philosophers in England in the 1950s to represent what they considered an ‘inferior’ style of philosophizing of ‘those’, ‘the other’, not like Anglo‐American analytic philosophers, those over there on the European continent and perhaps the few right in the midst of the English world who think like them (Glendinning, 2005). Richard Kearney, therefore, says that “the term ‘continental’ philosophy was coined not by European thinkers themselves but by academic philosophy departments in the Anglo‐American world eager to differentiate it from analytic thought.” (1994: p. 1). So, this was a glaring case of differentia‐politics about philosophy in which Anglo‐American philosophy prided itself on its analytic tradition in philosophy and constructed a difference of itself from European philosophical thought in a manner that empowers it and gives it control over the theoretical philosophical space.

The robustness of Continental philosophy as an umbrella term for many philosophical theories such as Marxism, Hegelianism, Idealism, Kantianism, Phenomenology, Existentialism, Hermeneutics and Structuralism/Poststructuralism, is responsible for the difficulty of providing a universal definition for its concerns beyond the geographical and historical definition attempted above. Yet, conversations and discourse over the centuries in Continental philosophy have had a significant impact on human society and discourses on issues of liberation, justice, autonomy, personhood and self, perception, existence, reason, gender, politics, interpretation, and understanding. It has 'exert[ed] a decisive impact on contemporary thought over the decades—an impact which exceeds the specialized discipline of academic philosophy and embraces such diverse fields as sociology, political science, literary theory, theology, art, history, feminism, and a variety of cultural studies.' (Kearney, 1994; p. 1). Of particular interest to us here is a somewhat consensual theorization of the person or the self in Continental philosophy and the impact it has had on healthcare, particularly in the West.

The self, the subject, or the person in continental philosophy is a rational, thinking, autonomous, individual subject, self or agent, distinct, separate or detached from objects and capable of providing grand universal theories and understanding of the world and things in it through reason, using approaches that are transcendental or non‐transcendental depending on the school of thought or movement in Continental philosophy. We immediately see the roots in Cartesian philosophy and how it becomes a fully fledged philosophy of the self in Kantian and Hegelian philosophy:

in the modern Cartesian and post‐Cartesian era, the ‘I think’ became the true substrate, as Descartes established in the first two of his ‘meditations’. Hence the term ‘subject’ came to designate the thinking ‘I’ in so far as this self becomes the new foundation for philosophical reflection. For Descartes, the ego as subject is what is certain, and the external world was rendered problematic. Post‐Cartesian thought endeavored to resolve this problem, and one sees in Kant and Hegel the attempt to have the subject encompass much more than a mere ‘inner’ sphere, and instead determine the whole of reality. With Kant, the subject thus becomes transcendental, which means that the subject is now the condition of possibility of objectivity itself, and no longer problematically cut off from it. Eventually in German Idealism, and in Hegel particularly, the subject is absolutized so as to become the totality of all that is as absolute Spirit (Raffoul, 2005; p. 562).

This understanding of the person thus prioritizes the self as the rational, conscious, autonomous embodied subject and often marginalizes and objectifies the other‐than‐the‐self. This disregard for the other became the basis for important critical thoughts about the self in 20th century Continental philosophy, a stage often represented as post‐dominant European and Anglo‐American thought. The writings and thoughts of prominent Continental philosophers in the 20th century such as Emmanuel Levinas, Martin Buber, Gabriel Marcel, Jacques Derrida and Michel Foucault on alterity, difference, and the self‐other relationship come to mind here. For example, Levinas (1998, 1999) theorizes the self's encounter with the other as an ethical responsibility, where “the feeling of responsibility for the other is not a rational choice but something that happens to you and that you experience as being chosen or ‘elected’ and that makes you unique, irreplaceable vis‐à‐vis the unique other. There is an ethical call to surrender to the other… One does not invite it or rationally accept it or find it justified or understand it: it just happens to one.” (Nooteboom, 2012; p. 162). Derrida too does a rigorous critique of dominant Western and Continental perspectives of the self, theorizing radical alterity in which, through deconstruction, there is a reversal of self‐privileging to the privileging of the other (Priest, 1994). Derrida has thus often faced the criticism that his deconstruction of the Western metaphysical understanding of the self to acknowledge the irreducibility of the other happens in a way that the absolute other increasingly resembles the absolute self, leading to a mere transfer of power rather than an absolute break with the metaphysical residues of logos (Bellou, 2013; p. 220). Also, Foucault's concept of the care of the self (1997) while an important shift from the conventional understanding of the self in Western and Continental thoughts in its acknowledgment of the subjectivity of the other remains an affirmation of the self and the other as embodied subjects, where the self needs to, first of all, care for its self its differences, freedom, and uniqueness. For as Foucault (1997: p. 287) puts it, 'Care for others should not be put before care for oneself. The care of the self is ethically prior in that the relationship with oneself is ontologically prior.' And we again find similar thoughts of the self as an embodied subject in the phenomenology of Maurice Merleau‐Ponty (1958).

Therefore, these understandings of the self in what might be called key phases of Continental philosophy—the Enlightenment phase (including the Cartesian, Kantian and Hegelian perspectives) and the post‐Enlightenment/postmodern phase (including the Levinean, Derridean and Foucauldian perspectives), although are quite different as the latter is a critique of the former, results in the long run in a phenomenology of the self and of the other as embodied, anatomical subjects and due to its emphasis on the lived experience of one's own body, sustains the Cartesian understanding of the person The extent to which these understanding of the person are at the foundation of Western healthcare and medicine is obvious in the literature on medical phenomenology (Carel, 2011; Rodriguez & Smith, 2018; Toombs, 2001) and medical anthropology (Good, 2010; Manderson et al., 2012). Healthcare professionals including nurses are taught phenomenological methods, such as the epoché, reduction or bracketing, selfhood, embodiment, and affectivity as effective ways of avoiding biases so that one may attend to the phenomena in an open and unprejudiced way (Fernandez, 2020). The Cartesian derived perspective of the person and the phenomenological understanding of the person are key features of medical anthropology and phenomenology courses (Jaye, 2004). Thus, the Continental philosophy of personhood has had and continues to have major implications for healthcare systems particularly in the West. It has embedded in healthcare the concept of the person as an embodied subject with an anatomical body and consciousness, a thinking self, replicating the Cartesian ‘*cogito, ergo sum*’. What needs care, nurturing, healing and wellbeing is this embodied subject, in part or in whole with each part dealt with quite distinctly. Western orthodox medicine has been built around this approach to healing and caring for the body and/or the mind. The care for the physiological and mental parts of the self in a manner that detaches one from the other was and is still largely constitutive of Western healthcare systems. More so, the ideal embodied subject as conceptualized in the Enlightenment phase of the tradition of Continental philosophy, built around deeply entrenched notions of autonomy, masculinity, and rationality in European philosophy since its inception in Greece, continues to dominate medicine and healthcare. Rationality, able‐bodied‐ness, and ideal subjectivity are often associated with masculinity and emotions, and sentimentality and fragility is associated with femininity. As discussed above, the post‐Enlightenment phase of Continental philosophy in the 20th century saw a critique of such ideal notions of the body, the subject, and the person, and led to the flourishing of discourse on alterity. Feminism, for example, emphasized how these ideals do a disservice to women's bodies, emotions, experience, embodiment, and situated knowledge. But autonomy, the embodied self or the embodied other remains central in dominant and critical perspectives in the Continental tradition.

### 3.2 The African philosophy of personhood and healthcare

African philosophy is also a difficult concept to define particularly as its existence in the academic field since the 1950s has been a problematic and highly debated one due to the denial of its existence by Western philosophy in the bid to sustain a differentia‐politics of philosophy quite akin to the one that existed between Continental and Anglo‐American philosophy. But it is an exercise in futility going into the nitty‐gritty of such existential debate on African philosophy as philosophy is a human experience shaped into its history in different places and times and by implication, African philosophy consists of the philosophical thoughts of African and African diasporic peoples, their ontological, epistemological, and ethical theories about human existence and critical reflections on lived‐experiences. The rich literature on African philosophy shows that its historical development emerges from (i) the rich, broad, and diverse indigenous African thought on matters considered philosophical dating as far back to the earlier stages of human civilization, and (ii) its colonial, postcolonial experience (Hallen, 2002; Kwame, 2017).

African philosophy is a rich and diverse system of thought, but sub‐Saharan African philosophies do enjoy much semblance in thoughts. A central and enduring framework for African philosophical thoughts that cut through the diverse thoughts of different African places is Afro‐communitarianism often represented in numerous literature with the Zulu word, Ubuntu. Ubuntu conveys the idea that a person is a person through other persons. “It is a Zulu/Xhosa word, with parallels in many other African languages, which is most directly translated into English as ‘humanness’. Its sense, however, is perhaps best conveyed by the Nguni expression ‘umuntu ngumuntu ngabantu’, which means ‘a person is a person through other people’.” (Bolden, 2014). Afro‐communitarianism is therefore first and foremost a personhood philosophy that holds that personhood is communally determined or produced and does not merely emerge from a solitary, rational, autonomous self. Ubuntu thus emphasizes that no one is fully a person or can achieve personhood independent of others by interdependence, solidarity, communal harmony, and ontological equilibrium as great goods. The South African Nobel Peace Laureate and anti‐apartheid and human rights activist, Desmund Tutu aptly captures this point in his book, *No Future without Forgiveness*, when he says,

Harmony, friendliness, community are great goods. Social harmony is for us the summum bonum – the greatest good. Anything that subverts or undermines this sought‐after good is to be avoided like the plague. Anger, resentment, lust for revenge, even success through aggressive competitiveness, are corrosive of this good. (Tutu, 1999).

Personhood, as conceived in Afro‐communitarianism, has two key interwoven dimensions, the ontological and the normative. The ontological dimension of personhood is akin to the embodied subject in Continental philosophy consisting of the anatomical body and consciousness (mental states) but goes beyond this to include non‐bodily, immaterial, or spiritual features. For example, in Yoruba philosophy, a person has both the physical, anatomical head (*ori ode*) and inner head (*ori inu*). While the physical, outer head is seen as consisting of vital organs for a person's survival such as the eyes and brain, the inner head is seen as the determinant of a person's existence and destiny. Thus both the material and immaterial dimensions of the head are vital to being a person (Lawal, 1985). But having these ontological features is not enough for a human being to be a person in African communities. The normative dimension must be fulfilled. A human being must be a member of a community and see herself only as being a person through relationships with other members of the community as embedded in the concept of Ubuntu. She must actively sustain community, promoting equilibrium, solidarity, harmony, humanness, and communal wellbeing.

The obvious implication of the African concept of personhood for healthcare in indigenous African communities is the emphasis on an inclusive approach to healing, caring, and wellbeing. The idea that good health and wellbeing are essentially dependent on relationships permeates African communities. Ubuntu, ‘a person is a person through other persons’, sends a strong message that my general well‐being, and my health, is dependent on you and our co‐existence and relationship. Frictions, discord, emphasis on individualism – my own wellbeing alone would result in ill‐health, metabolic and social imbalances not just for myself but also for others. This is why African indigenous healthcare systems do not only care for, or focus on nurturing or healing, the body and the mind, they also nurture, heal and care for non‐bodily features of the human being and for mending and building relationships and inhibiting imbalances; this is palpable in African communities which is permeated with healing related rituals, rites, and activities.

## 4 PHILOSOPHIES OF PERSONHOOD AND IMPACT ON HEALTHCARE

In the two philosophies, we have examined and their conceptions of personhood, there is an inherent hegemony at work, the authoritarian, God's‐eye perspective as to who a person ought to be. In the Continental philosophical perspective, the autonomous embodied subject, often presented as the ‘healthy white able‐bodied rational male’ still reigns, influencing healthcare policies, best practices, and the attitude of healthcare professionals including nurses to patients. In the African philosophical perspective, the community‐defined person who has fulfilled both the ontological and normative requirements of personhood reigns, informing healthcare best practices and the attitude of healthcare professionals. These hegemonies do exactly what they are meant to do: they become preponderant, having a superior and domineering force and influence on healthcare systems. More important, they breed exclusion and do not allow for the inclusion and thriving of different perspectives at the same time in medical practices.

Exclusion happens in the denial, non‐recognition, or misrecognition of legitimate ontic and normative differences and the politics of universalization of, or sustained and often successful attempt to universalize, a particular ontic and normative perspective as the ‘true’ and ‘objective’ one over and above others. Within the Continental perspective, any understanding and lived realities of personhood that do not fit within the hegemonic and prevalent understanding as embodied subject faces the danger of being deliberately or non‐deliberately excluded from the mainstream and conventional healthcare systems. The prevalent understanding of the body and mind trivializes the very important situatedness of bodily or corporeal manifestations and experiences. The situatedness of the corporeality of the female body, the non‐Western body is still largely excluded from healthcare systems in Continental and Western systems. For example, Premenstrual Dysphoric Disorder was recognized only in May 2019 by World Health Organisation as a uniquely feminine health problem; and the exclusively white bodily images in conventional medical texts inhibit the understanding of non‐white bodies. This raises the question about who is pictured in the idea of an embodied subject, an autonomous self in healthcare and medicine.

Within the African perspective, not all ‘human beings’ fulfill the ontological and normative requirements of being persons. For this reason, they are treated as sub‐humans and non‐persons. Persons with disabilities such as persons with albinism, persons with epilepsy, persons with angular kyphosis, and persons with different types of mental health issues as well as ‘queer’ persons (understood in the sense of differing or weird in some way from what is usual or normal remembering for example, the killing of twins in several Nigerian communities for their queerness) fit this profile and are thus treated as sub‐humans. This obviously excludes such persons from enjoying full healthcare benefits within African communities (Imafidon, 2017, 2019, 2021). The impact of these on healthcare systems is quite obvious. Hegemonic conceptions of personhood results in a poverty of knowledge about the multi‐layered ontic and normative nature of personhood. It results in exclusion and discrimination of persons who do not fit within accepted models in specific places and this implies minimal healing, care, and nurturing for such persons, considering how important healthcare systems are to our general wellbeing.

An interesting parallel is the conception of a nurse‐person in African places and how this impacts male nurses. Nursing is essentially seen as caring and nurturing in African communities and the African understanding of care is intrinsically linked to motherhood and maternal tenderness. Imafidon (2018: p. 171) explains that,

… the African feminine conception of care revolves around the concept of motherhood understood strictly as maternal tenderness and affection toward the one cared‐for. It involves an ontic drive to promote the wellbeing of the one cared‐for. African women instinctively and intuitively know that they are mothers; they act and consistently work toward fulfilling their motherly roles and train younger females on how to do so. It is often assumed that motherhood for African women is directed only to biological children. An African mother is not only a mother to her children, but to her brothers, sisters, father, mother, friends, relatives, and even the environment. Her maternal tenderness and affection and the ontic drive to promote wellbeing is extended to the whole community, both human and nonhuman.

With specific reference to indigenous African healthcare, African women, not men, are the ones mostly allowed to be indigenous nurses and midwives carrying out their gender‐defined responsibilities of caring for and nurturing people and the community at large. Hence, the nurse‐person is feminine in the African understanding. This explains why if a male in an African community today decides to study or practice nursing, he is likely to face stigmatization and even ridicule (Achora, 2016; Kalemba, 2019). This further reiterates how conceptions of personhood in general or specific forms of persons such as nurses in particular may inhibit diversity, inclusion, and equality in access to healthcare.

## 5 CONCLUDING THOUGHTS: TOWARD AN ECOLOGY OF MEDICAL BITS OF KNOWLEDGE

The French writer, feminist and existentialist philosophy, Simone de Beauvoir rightly says in The Second Sex (1949: p. 143) that ‘Representation of the world [in general and of personhood in particular] like the world itself, is the work of men; they describe it from their own point of view, which they confuse with absolute truth’. Any philosophy of the person is always a perspective and thus, always incomplete knowledge of the person needing revision and improvement. Our lived horizons are more than ever shaped by globally diverse bits of knowledge and perspectives. Healthcare systems today globally cater to a diverse body of persons that have different situated healthcare needs and have emerged from different places and spaces and different conceptions of personhood. The recognition and understanding of these differences and the decolonization of healthcare spaces are extremely important if stakeholders including nurses are to genuinely succeed in caring for persons. This would involve taking situated personhood and medical knowledge seriously to support person‐centered healthcare, decolonizing and indeed, demasculinizing healthcare systems by taking a feminine and cultural understanding of health, caring, and wellbeing seriously and, by implication, recognizing authentic ontic and normative differences in healthcare. Such decolonization and demasculinization of healthcare systems can only happen through what Boaventura de Sausa Santos calls the ecology of knowledge (2007). In his words,

…an ecology of knowledge… is premised upon the idea of the epistemological diversity of the world, the recognition of the existence of a plurality of knowledges beyond scientific knowledge. This implies renouncing any general epistemology. Throughout the world, not only are there very diverse forms of knowledge of matter, society, life, and spirit, but also many and diverse concepts of what counts as knowledge and the criteria that might be used to validate it (2007: 67).

Bearing this in mind and with particular reference to medical knowledge in general and of the person in particular, the ecology of medical knowledge exposes the medical field to a robust and diverse approach to understanding the person, healing, caring, nurturing, and wellbeing. It allows health workers including nurses to acknowledge the fallibility and dynamism of medical knowledge, that every single knowledge claim is incomplete and that an interrelationship of the many medical pieces of knowledge leads to a better and more effective healthcare system.

## CONFLICT OF INTEREST

The author declares no conflict of interest.

## Data Availability

Data sharing not applicable to this article as no datasets were generated or analysed during the current study.
